# Influence of Maternal Dietary Protein during Late Gestation on Performance of Black Bengal Does and Their Kids

**DOI:** 10.3390/ani14192783

**Published:** 2024-09-26

**Authors:** Md Sayaduzzaman Arafath, Mahadi Hasan, Jakia Sultana, Md Hasanur Alam, Asma Khatun, Mohammad Moniruzzaman

**Affiliations:** Department of Animal Science, Bangladesh Agricultural University, Mymensingh 2202, Bangladesh

**Keywords:** Black Bengal goat, blood parameters, carcass characteristics, dietary protein, growth, *H-FABP* gene, late pregnancy, milk yield

## Abstract

**Simple Summary:**

The goat is used as a tool of poverty reduction at the village level in developing countries. The Black Bengal is a popular goat breed in Bangladesh and the eastern part of India. They have high prolificacy and skin quality and delicious meat. A high level of kid mortality and poor growth rate represent a significant barrier to increasing the productivity of Black Bengal goats. Low birth weight and insufficient milk yield of dams are responsible for the poor growth and high mortality of these kids. It is considered that maternal nutrition during pregnancy plays an important role in the birth weight of kids and the lactation performance of dams. In the present study, the effects of three levels (18, 14, and 10 percent) of dietary crude protein (CP) during late pregnancy on the growth performance of dams and kids, birth weight of kids, and milk production of does, as well as blood parameters, survivability rate, carcass characteristics and meat composition of kids, and expression of the *H-FABP* gene related to muscle fat content, were investigated. The results show that maternal dietary protein during late pregnancy enhances the milk production of does and the growth and carcass characteristics of Black Bengal kids.

**Abstract:**

The present study aimed to elucidate the effect of different levels of dietary protein during late pregnancy on the performance of Black Bengal does and their kids. Twelve does were divided into three groups, with four in each, and three diets, i.e., high protein (18% CP), medium protein (14% CP), and low protein (10% CP) were supplied for 50 days, commencing from 100 days post-coitum to parturition. During the first 100 days of pregnancy, uniform rations with similar ingredients were provided to fulfill the nutrient requirements depending on the live weight of does. All three diets were isocaloric (10.0 MJ/kg DM). Data were subjected to one-way ANOVA, and the significance of the difference among means was determined by Duncan’s Multiple Range Test (DMRT). The main effects of diet and sex, as well as their interaction, were analyzed by two-way ANOVA by using the GLM procedure. The relative expression values of qPCR were calculated by using the 2^−ΔΔCt^ analysis method. Live weight gain was significantly (*p* < 0.05) higher in high-protein-fed dams than other groups during the experimental period. The milk yield of does was significantly (*p* < 0.05) higher in high-protein-fed goats than in the low-protein group. The lactation length of does was significantly (*p* < 0.05) higher in the high- and medium-protein-fed does than in the low-protein-fed does. The duration of post-partum anestrus of does was significantly (*p* < 0.05) higher in the low-protein-fed dams than in the high-protein group. The birth weight of kids tended to be higher in the high-protein group but did not differ significantly among the treatment groups. In male kids, weaning weight, final weight, live weight gain, and average daily gain were significantly (*p* < 0.05) higher than in female kids. Weaning weight was higher (*p* < 0.05) in kids of the high-protein-fed does than the low-protein group. Final weight and live weight gain were significantly (*p* < 0.05) higher in kids of the high-protein-fed does than in the low-protein-fed group. On the other hand, average daily gain was significantly (*p* < 0.05) higher in kids of the high- and medium-protein-fed does than the low-protein group. The average body length and wither height of kids at the 32nd week was significantly (*p* < 0.05) higher in kids of high-protein-fed does than those of the low-protein-fed group. The average heart girth of kids at the 32nd week was significantly (*p* < 0.05) higher in kids of high-protein-fed does than the medium- and low-protein groups. The survival rate of kids was higher in the medium- and high-protein-fed does than in low-protein group. Hot carcass weight and ether extract content of meat were significantly (*p* < 0.05) higher in the high-protein group than in the other groups. The dressing percentage was significantly (*p* < 0.05) higher in the kids of high-protein-fed does than low-protein-fed goats. The expression of the *H-FABP* gene was significantly (*p* < 0.05) higher in kids of high-protein-fed does than those of the medium- and low-protein groups. In conclusion, maternal dietary protein levels positively influences the production performance of Black Bengal does and their kids.

## 1. Introduction

The goat is one of the earliest domesticated animals and has been associated with humans for around 10,000 years [1]. Due to their adaptability to different environmental and climatic conditions, they are dispersed all over the world [2]. Goats are the most beneficial animals in the world, providing meat, milk, fiber, fertilizer, and draft power [3]. They play an important role in generating employment, income, capital storage and improving household nutrition. The goat is considered the poor man’s cow, as they are used as a tool of poverty reduction at the village level. The Black Bengal is a popular goat breed in Bangladesh and the eastern part of India. They have high prolificacy and skin quality and delicious meat [4]. A high level of kid mortality represents a significant barrier to increasing the productivity of Black Bengal goats. Low birth weight, insufficient milk of dams just after kidding, and poor husbandry practices are mainly responsible for higher kid mortality in Black Bengal goats [4]. It is thought that maternal nutrition during pregnancy plays an important role in the wellbeing of goats and their newborn kids. Inadequate nutrition during gestation can delay the onset of lactogenesis and reduce colostrum and milk production [5].

Late gestation is the period when nutritional demands are the highest for maintaining the dam’s health and mammary development for high milk production, and many of these requirements are met by adaptive physiological changes that occur during gestation. It has been reported that 80% of fetal growth takes place in the last trimester of the pregnancy, requiring an increase in nutritional requirements during this period [6]. Concentrate feeding for pregnant does and ewes during this advanced phase of pregnancy promotes the growth performance of both the mother and the growing fetus [7].

A diet containing 7% dietary crude protein is needed for normal rumen bacterial growth and function in goats. Diets containing less than 7% result in a depression in both forage intake and digestibility [8]. The recommended crude protein requirement for goats during late pregnancy and lactation ranges from 11% to 14% CP for does [9]. The traditional system of goat rearing in Bangladesh cannot fulfill these nutrient requirements to sustain proper growth and reproductive performance, resulting in severe economic losses [10]. The effects of dietary protein during late pregnancy on the growth and performance of Black Bengal does and their kids are not well known. Therefore, the present experiment was designed to shed light on the effects of different levels of dietary protein in late pregnancy on the growth and milk production of Black Bengal does, as well as the birth weight, growth rate, body measurements (heart girth, height at wither, and body length), blood parameters, survivability rate, carcass characteristics, and meat composition of their kids.

## 2. Materials and Methods

### 2.1. Place of Experiment

The experiment was conducted at the Goat, Sheep and Horse farm of the Department of Animal Science, Bangladesh Agricultural University, Mymensingh, Bangladesh.

### 2.2. Housing and Management Practices

Pure Black Bengal does (1.5 years old) of second parity (*n* = 12) were housed individually in 12 separate pens. Each pen covered an area of 1.38 square meters. The house was well ventilated. In order to protect animals from rain and wind, gunny bags were hung over the ventilator. Phenyl was used as the antiseptic to clean the floor, feeder, and water trough. Ear tags were used to identify the animals. Goats were dewormed by using an anthelmintic drug (Lezol-4; FnF Pharmaceuticals Ltd., Dhaka, Bangladesh) according to the instructions of the manufacturer following the assessment of fecal egg counts.

### 2.3. Design of Experiments

Goats were divided into three groups with each having four (4) does. Three levels (10%, 14%, and 18%) of dietary crude protein (CP) were supplied during late pregnancy (100–150 d). After showing estrus, does were mated with a pure Black Bengal buck, and the day of service was considered d 0. This experiment was conducted with goats of pure Black Bengal breed. Usually, the goats of this breed exhibit similarity in traits related to production and physiological parameters [11]. Therefore, individual variations among these goats were smaller than other goat populations.

### 2.4. Formulation of Experimental Diets

Three diets were formulated by using locally available feed ingredients, including German grass, wheat bran, maize crushed, soybean meal, DCP (dicalcium phosphate), and common salt. The medium protein diet was formulated to contain 14% crude protein to fulfill their protein requirement. The other protein levels were high (18% crude protein) and low (10% crude protein). The ingredients and chemical composition of the formulated experimental diets are presented in Table 1.

### 2.5. Feeding of Does

The diet was adjusted for the live weight change of the animals under treatment every seven days. Requirements for energy and protein were calculated depending on the body weight of does during late pregnancy. Protein requirement was calculated as 6.97 × Live weight^0.75^ g CP/day [16,17]. The required amount of concentrate feed for an individual doe was divided into two equal parts and supplied twice daily, at 0800 and 1600 h. Green grass was supplied 3 times daily. Fresh water was supplied ad libitum. Feed refusals were weighed and recorded every morning before replenishing feed. 

### 2.6. Kid Management

Newborn kids (*n* = 21), [high (*n* = 7, with males = 3 and females = 4), medium (*n* = 6, with males = 3 and females = 3), and low (*n* = 8, with males = 3 and females = 5)] were kept with their dams, and a dry cloth was used to gently rub the nostrils to remove mucous. The suckling of milk of each kid from their mother was checked closely. After one month, kids were offered succulent roughages and fine concentrate mixtures.

### 2.7. Live Weight Measurement

Does and their kids were weighed individually weekly by using an electronic balance (RFL, Dhaka, Bangladesh). Weighing was performed at 0900 h before offering feed and water, and data were recorded individually.

### 2.8. Milk Production Record

The milk yield of individual does was measured weekly. The kids were separated from their mother for a period of 3 h (from 0900 to 1200 h or from 1500 to 1800 h). Kids were then allowed to suckle milk from their mother and weighed before and after suckling to determine their milk intake, with care taken to adjust for any urine output over this period. The differences between these two weights were considered the amount of milk consumed by the kid. This value of milk consumption was then multiplied by 8 to estimate the amount of milk produced in a day (24 h).

### 2.9. Body Measurements of Kids

A measuring tape was used each week to record the kids’ height at wither, heart girth, and body length. The distance between the base of the ear and the base of the tail was assessed as body length. The wither height was measured from the highest point of the wither to the ground with the animal standing erect on all four hooves, all legs parallel to the ground, and head in an upright position. Heart girth was assessed by measuring the circumference around the chest, directly posterior to the front legs and wither.

### 2.10. Blood Parameter Analysis of Kids

Blood was collected from the jugular vein of kids before feeding in the morning. Immediately after collection, the blood was transferred to blood clot activator tubes (LEVRAM^TM^, Mumbai, India), and serum was separated by micropipettes. Blood sera were stored at −20 °C until used for biochemical analysis. Commercial diagnosis kits (Labkit, Barcelona, Spain) were used for the determination of total protein, albumin, glucose, globulin, and triglyceride following the manufacturer’s protocol either by the kinetic or endpoint method as appropriate. All the tests were performed on a semiautomatic biochemistry analyzer (Dymind, Shenzhen, China) at the desired wavelength.

### 2.11. Carcass Parameters and Meat Composition

After weaning, three male kids from each group were selected randomly and slaughtered by the Halal method. Kids were fasted for 24 h before slaughter. The hot carcass weight, dressing percentage, and offal weight were recorded. The dressing percentage was calculated by the following formula:

Dressing percentage (%) = (hot carcass weight/live weight) × 100.

After slaughter, the *longissimus dorsi* muscle of each kid, located between the 12th and 13th ribs, was sampled to determine the nutrient content of the meat. The standard protocols were followed for the assay of dry matter, crude protein, ether extract, and crude ash contents [18].

### 2.12. Expression of H-FABP Gene

Muscle samples (*n* = 3 per group) were initially ground by using a TissueLyser II (QIAGEN, Hilden, Germany) at 25 Hz 30 frequency/sec for 10 min to separate nucleic acid. Total RNA was isolated from nucleic acid samples by using the Monarch Total RNA Miniprep Kit (New England BioLabs Inc., Ipswich, MA, USA; lot No. 10097588) according to the manufacturer’s protocol. The concentration and purity of the extracted RNA was checked by using a MULTISKAN™ SkyHigh Microplate Spectrophotometer (Thermoscientific, Waltham, MA, USA; catalog number A51119500C) to calculate the RNA concentration (ng/μL; A260/A280 ratio and A260/A230 ratio for each sample). The cDNA was synthesized with a high-capacity Thermoscientific Verso cDNA Synthesis Kit (Applied Biosystem, Bedford, MA, USA; lot No. 2871399; REF. AB-1453/A) according to the manufacturer’s protocol. The concentration and purity of the extracted cDNA was checked by using a MULTISKAN™ SkyHigh Microplate Spectrophotometer (Thermoscientific). To assess *H-FABP* gene expression, real-time quantitative PCR was performed by using PowerUp™ SYBR™ Green Master Mix (Applied Biosystems, Bedford, MA, USA; lot No. 01271938; REF. A25741). The RT-qPCR conditions for *H-FABP* were 50 °C for 10 min, 95 °C for 2 min followed by 40 cycles of 95 °C for 15 s, 58 °C for 30 s, and 72 °C for 1 min. The melting temperature was set to 95 °C for 15 s, 60 °C for 1 min, and 95 °C for 15 s for qPCR. The primer for *H-FABP* was used as the positive marker where the *β-actin* gene was used as an internal reference for normalization for *H-FABP* mRNA expression. The primer sequences of the *H-FABP* and *β-actin* genes are presented in Table 2.

### 2.13. Statistical Analysis

All values were expressed as means ± SEs (standard errors). All data were subjected to one-way ANOVA, and the significance of the difference among the means was determined by Duncan’s Multiple Range Test (DMRT). The main effects of diet and sex, as well as their interaction, were analyzed by two-way ANOVA by using the GLM procedure. A probability of less than 0.05 was considered statistically significant. All statistical analyses were conducted by using IBM SPSS Statistics 20. The data from the qPCR experiment were calculated by the ΔΔCt (critical threshold) method, with *β-actin* serving as the internal control. The 2^−ΔΔCt^ analysis method was used to calculate the relative expression values for the qPCR assay.

## 3. Results

### 3.1. Live Weight Changes of Does

Before parturition, the average initial live weight of does in different protein feed groups was similar (Table 3). The final live weight of does did not differ significantly among the treatment groups (*p* > 0.05). However, the average live weight gain of the high-protein-fed (18% CP) does was significantly (*p* < 0.05) higher than that of the other groups (14% and 10% CP). The average daily feed intake (ADFI) tended to be higher in the high-protein-fed (18% CP) does than in the medium-and low-protein-fed groups, although the values did not differ significantly (*p* > 0.05). A negligible quantity of refused feed was obtained every day from all treatment groups. Thus, the actual intake of nutrients was the same as the actual composition listed in Table 1.

**Figure 1:**
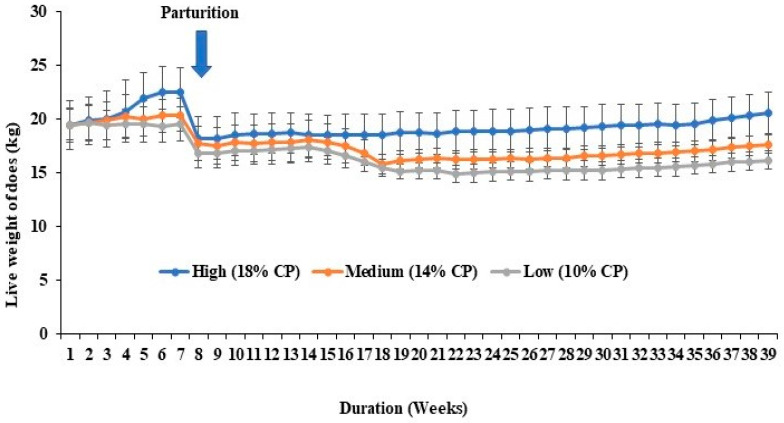
Live weight changes of does (*n* = 4 per group). The line charts represent the average live weight changes of does (kg) from different groups every week. The error bars represent the standard errors of the mean (SEMs) from the replication of the experiments.

The live weight of does in the three treatment groups over the course of the experiment are shown in Figure 1. No significant differences in the live weight of does among the three treatment groups were found. The live weight of does tended to be higher in the high-protein-fed (18% CP) does than in the medium- and low-protein-fed groups before parturition. After parturition, the live weight of does tended to decrease in the medium- (14% CP) and low-protein-fed (10% CP) groups but remained constant in the high-protein (18% CP) group. 

### 3.2. Milk Production Performance of Does

**Figure 2:**
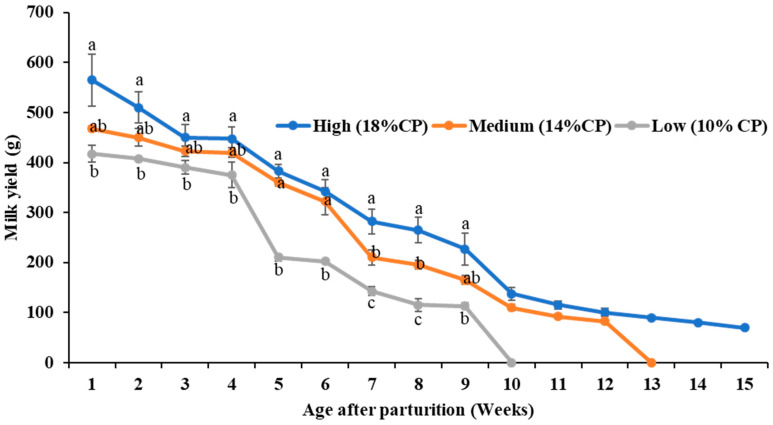
Milk yield of does (*n* = 4 per group). The line charts represent the average milk yield of does (g) from different groups every week after parturition. The error bars represent the standard errors of the mean (SEMs) from the replication of the experiments. ^a,b,c^ indicate significant differences among the three groups.

Milk yield was significantly (*p* < 0.05) higher from the 1st to the 4th week in the high- protein-fed (18% CP) does compared with the low-protein (10% CP) group (Figure 2). During the 5th and 6th weeks, milk production was significantly (*p* < 0.05) lower in the 10% CP fed does compared with the other groups. During the 7th and 8th weeks, milk production from the 18% CP group outperformed the others significantly (*p* < 0.05), while the 14% CP group also outperformed the 10% CP group significantly (*p* < 0.05) at the same time. The significant differences between the 18% CP and 10% CP persisted until the 9th week. 

### 3.3. Lactation Length, Post-Partum Anestrus of Does and Birth Type, and Sex and Birth Weight of Kids

Litter size, birth type, sex, average lactation length, the post-partum anestrus of does and the birth weight of kids are shown in Table 4. The litter size did not differ significantly (*p* < 0.05) among high- (18% CP), medium- (14% CP), and low-protein (10% CP)-fed goats. Medium-protein-fed (14% CP) does delivered the same number of single and twin kids. The 10% and 18% CP-fed does provided the highest twinning rates, 100 and 75%, respectively. No triplets were found in this experiment. The 14 and 18% CP does yielded more male kids than the 10% CP group, while the latter group provided more female kids. The birth weight of male and female kids did not differ significantly (*p >* 0.05) within the treatment groups. There was no effect (*p* > 0.05) of litter size on the birth weight of kids. The lactation length was significantly (*p* < 0.05) higher in the high-protein-fed (18% CP) goats. Lactation length was significantly (*p* < 0.05) longer in the medium-protein-fed (14% CP) does than in the 10% CP group. 

The post-partum anestrus was significantly (*p* < 0.05) shorter in the 18% CP group (86.50 ± 0.50 d) than in the 10% CP group (89.67 ± 1.45 d). The mean birth weight tended to be higher in kids from the 18% CP group compared with the others (*p* > 0.05).

### 3.4. Growth Performance of Kids

**Figure 3:**
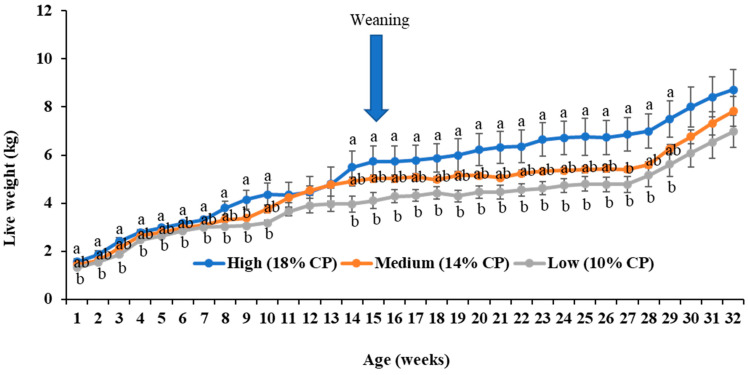
Effects of maternal dietary protein levels on live weight changes of kids (*n* = 5 per group). The line charts represent the average body weight changes of kids (kg) from different groups every week. The error bars represent the standard errors of the mean (SEMs) from the replication of the experiments. ^a,b^ indicate significant differences among the three groups.

Although the live weight of kids from the high-protein-fed (18% CP) does often exceeded significantly than that of the low-protein-fed (10% CP) group up to week 10, there was a major significant (*p* < 0.05) divergence in live weight gain between these groups from the 14th to the 32nd week (Figure 3). Weaning weight, final live weight, live weight gain, and average daily gain were all significantly (*p* < 0.05) higher in the high-protein-fed (18% CP) group than in the low-protein-fed (10% CP) group, with intermediate values being recorded for the medium-protein-fed (14% CP) group with varying levels of significance (Table 5). The same significant (*p* < 0.05) differences were observed between the male and female kids. The interaction effects of diet and sex were not significant (*p* > 0.05) on any of these parameters (Table 5).

### 3.5. Body Parameters of Kids

**Figure 4:**
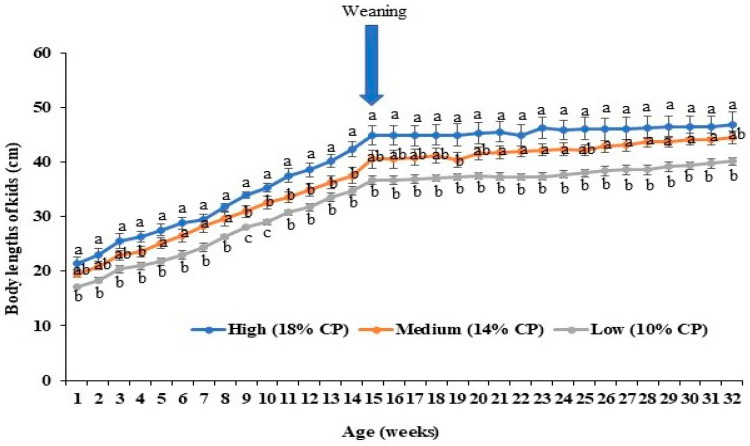
Effects of maternal dietary protein levels on body length of kids (*n* = 5 per group). The line charts represent the average body lengths changes of kids (cm) from different groups every week. The error bars represent standard errors of the mean (SEMs) from the replication of the experiments. ^a,b,c^ indicate significant differences among three groups.

The body length of kids from the 1st week to the 32nd week are shown in Figure 4. Throughout the whole growth cycle, kids from the high-protein-fed (18% CP) does were significantly longer than those from the low-protein-fed (10% CP) group (Figure 4). Values for the kids which were the product of the 14% CP fed does were intermediate between these, with variable levels of significance across time points.

**Figure 5:**
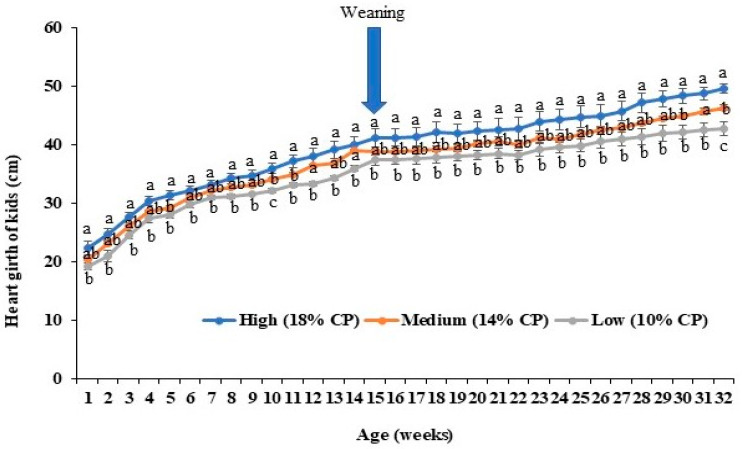
Effects of maternal dietary protein levels on heart girths of kids (*n* = 5 per group). The line charts represent the average heart girths of kids (cm) from different groups every week. The error bars represent standard errors of the mean (SEMs) from the replication of the experiments. ^a,b,c^ indicate significant differences among three groups.

Similar trends were observed for measurements of heart girth. Again, kids from the high-protein-fed (18% CP) does displayed the highest heart girths, with all values across the growth profile being significantly higher than values from kids obtained from the low-protein-fed (10% CP) group (Figure 5). Again, the medium-protein-fed (14% CP) protein diet generated kids with intermediate values for all time points of the growth cycle with differences from the other two groups displaying variable levels of significance (Figure 5). 

**Figure 6:**
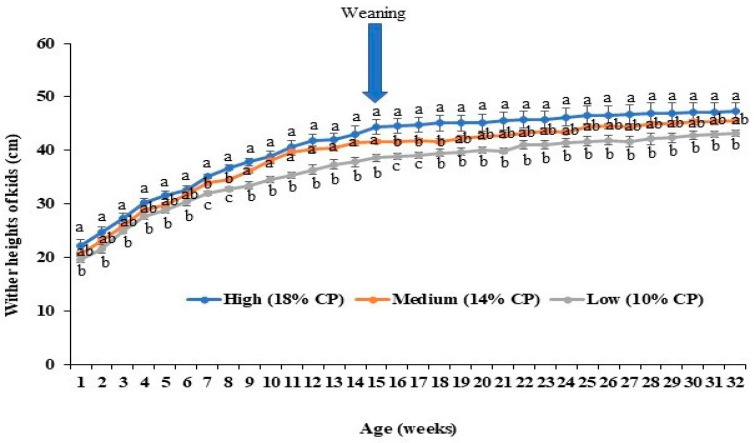
Effects of maternal dietary protein levels on wither heights of kids (*n* = 5 per group). The line charts represent the average wither heights of kids (cm) from different groups every week. The error bars represent standard errors of the mean (SEMs) from the replication of the experiments. ^a,b,c^ indicate significant differences among three groups.

Not surprisingly, very similar trends were observed for wither heights, with significant differences between the highest and lowest protein diets and intermediate values for the 14% CP diet, again with variable levels of significance across the growth profile (Figure 6). 

### 3.6. Blood Parameter Analysis in Kids

The effects of maternal dietary protein levels on blood parameters of kids during the pre-weaning (at the 15th week) and post-weaning (at the 17th week) periods are shown in Table 6 and Table 7, respectively. Serum glucose, total protein, albumin, globulin, and triglyceride concentrations tended to be higher in kids from high-protein-fed (18% CP) does than for the kids from the medium- (14% CP) and low-protein-fed (10% CP) groups but were not significant (*p* > 0.05). This trend was reversed for circulating triglyceride concentrations in both the pre- and post-weaning periods (Table 7).

### 3.7. Survivability of Kids

The survivability percentages of kids during the experimental period are shown in Table 8. The survivability of kids was higher in the kids from the medium-protein-fed (14% CP) does than for the other two levels.

### 3.8. Carcass Characteristics and Meat Composition

The effects of maternal dietary protein levels on carcass characteristics of kids are shown in Table 9. The slaughter weight and hot carcass weight were significantly (*p* < 0.05) higher in kids that were the product of the high-protein (18% CP) diet than for the lower-CP diets. However, the values did not differ significantly between medium- and low-protein groups. The dressing percentage was significantly (*p* < 0.05) higher in kids from the high-protein group than those from the lower-protein-fed groups. No significant (*p* > 0.05) difference was found in offal weight among the treatment groups. As shown in Table 10, the fat (ether extract) content of the meat was significantly (*p* < 0.05) higher in kids from the high-protein-fed does than those from the lower protein groups. Maternal dietary protein did not influence the dry matter, crude protein, and ash contents of the *longissimus* muscle of the kids (*p* > 0.05).

### 3.9. Expression of Heart Fatty Acid-Binding Protein (H-FABP) mRNA 

**Figure 7:**
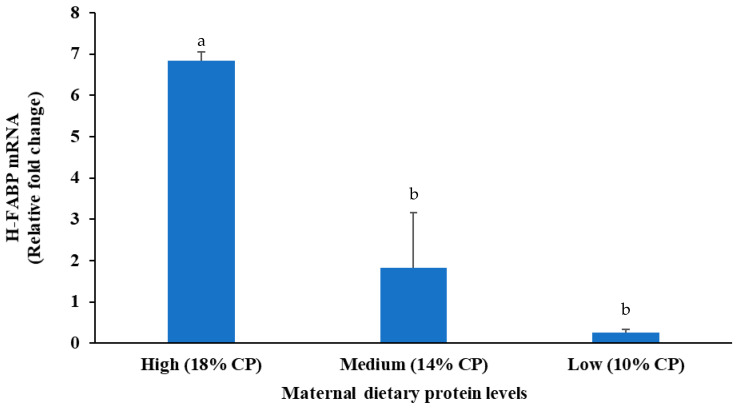
Effects of maternal dietary protein levels on the expression of the heart fatty acid-binding protein (*H-FABP*) gene related to fat content in meat (*n* = 3 per group). The bars represent the average fold changes calculated by using the ΔΔ critical threshold method. The error bars represent standard errors of the mean (SEMs) from the replication of the experiments. ^a,b^ indicate significant differences among three groups.

The effects of maternal dietary protein on the expression of the heart fatty acid-binding protein (*H-FABP*) gene are shown in Figure 7. The expression level of the *H-FABP* gene was assessed as a threshold cycle value for muscle samples of kids. There was a significant difference between the kids from does consuming the different protein diets. Kids from the high-protein-fed (18% CP) does showed significantly (*p* < 0.05) higher *H-FABP* gene expression levels than those from the medium- (14% CP) and low-protein (10% CP) groups. However, the values did not differ significantly between medium- and low-protein groups.

## 4. Discussion

In the present study, different levels of dietary protein were supplied to Black Bengal goats during late pregnancy to examine the effects of maternal dietary protein on the performances of does and their kids. The result showed that the average live weight gain of high-dietary-protein-fed (18% CP) does was higher than that of low-protein-fed (10% CP) does. A similar result was obtained by Sahlu et al. [19], who used Alpine does to report that CP intake positively increased live weight gain. Phengvichith and Ledin [20] reported that high protein and energy increased the live weight and daily weight gain in female does. It was also reported that, the lowest CP level (8.7%) diet offered may have limited the development and activity of rumen microbes, whereas the higher CP levels most likely stimulated microbial activity, organic matter fermentation, and microbial protein synthesis [21]. It is thought that higher levels of dietary protein stimulate microbial activity, organic matter fermentation, and microbial protein synthesis. This is consistent with the superior performance of kids derived from the high-protein-fed (18% CP) does in the present study.

In this study, milk yield was higher from the high-protein-fed does than the low-protein group. A similar result was reported by Ahmed et al. [22], who conducted an experiment with Barki ewes and reported that total milk yield was higher in medium- (13% CP) and high-protein (15% CP)-fed ewes during pregnancy than the low-protein-fed (11% CP) group. Sahlu et al. [23] reported that milk production increased quadratically with pre-partum dietary protein concentration in Alpine does. It was also reported that supplementary feeding improved milk yield in Kilis goats [24]. This is consistent with our results, according to which the duration of lactation was related closely to the dietary protein level of does. Similarly, Saha et al. [25] reported that the supplementary feeding of concentrate increased lactation length in Black Bengal goats. Higher protein diets have been associated with body fat mobilization to promote the production of milk in Holstein cows [26]. Similarly, ewes (Leicester × Scottish Blackface) fed a lower protein diet (65 g/68-kg ewe per day) have been reported to produce less milk, presumably because of the limitation in dietary protein [27]. Low-protein diets have been shown to limit not only the flow of non-ammonia nitrogen to the small intestine but also rumen fermentation and carbohydrate digestibility [28]. If the diet limits the availability of ammonia nitrogen, amino acids, and peptides to rumen microorganisms, dry matter digestibility might decrease, which, as a consequence, reduces the availability of digestible energy [28]. Thus, it is assumed that high-protein diets might increase the availability of ammonia nitrogen, amino acids, and peptides in the rumen, which might ultimately increase microbial protein synthesis. This most likely explains the higher milk production in does consuming the high-protein-diet in the present experiment.

Our study also showed that the post-partum anestrus period was shorter in the high-protein-fed (18% CP) does than the low-protein-fed (10% CP) group. Dunn and Kaltenbach [29] revealed that undernutrition increases the incidence of anestrus in primiparous ewes, while feeding high-protein diets shortens the post-partum interval in the dairy cow. De Feu et al. [30] stated that cows offered high dietary energy displayed a significantly shorter post-partum heat period than the control group. In contrast, Butler and Smith [31] revealed that negative energy balance caused delayed ovarian activity associated with decreased LH secretion. In the present study, the reason for a shortening post-partum anestrus period in high-protein-fed goats requires further study. However, it is assumed that a high-protein-diet might increase ovarian activity and associated hormone secretion in our goats, which, in turn, might help explain the short post-partum anestrus. 

The weaning weight, final weight, live weight gain, and average daily gain of kids was higher in kids from the high-protein-fed (18% CP) does than the low-protein-fed (10% CP) group. Shahjalal et al. [10] fed Black Bengal goats diets containing 16.9 and 20.3% CP and reported that increasing dietary protein resulted in a higher growth rate. Negesse et al. [21] conducted a similar trial with Saanen goats, reporting that kids fed a 17.6% CP diet gained more weight than those offered 14.4%, 11.4%, or 8.7% CP diets. In a further study, additional or supplemental feeding during lactation increased birth and weaning weights of Kilis goat kids irrespective of parity, due to the increased milk yield of mothers [24]. Similarly, in our experiment, the weaning weight, final weight, live weight gain, and average daily gain of kids from high-protein-fed goats were higher, possibly due to the consumption of more milk from their mothers. Protein is an essential nutrient for animal growth and development; thus, a sufficient protein supply is a crucial factor for normal growth when provided in balance with increased dietary energy. The pre-partum diets of dams are known to influence the postnatal growth and average daily gain of offspring [32]. Skeletal muscle mass is largely determined by the number and the size of muscle fibers. Muscle fibers are formed exclusively during the prenatal stage, and there is no further net increase in the number of these fibers post-partum [33]. It is assumed that the high-protein maternal diets fed during pregnancy in the present study might have increased the number of muscle fibers found in the kids, leading to higher muscle mass, growth rate, and finally slaughter weight in the kids. According to this hypothesis, this then led to higher body length, wither height, and heart girth in kids from the high-protein-fed groups. The wither height and heart girth of Sistani goats were correlated to the live weight of kids, the volume of milk consumed, and all other measured characteristics [32]. Similarly, Dorantes-Coronado et al. [34] found a high correlation between heart girth and body length with the live weight of goats. In the present experiment, the milk production of does was higher in high-protein-fed groups, so kids of high-protein-fed group consumed more milk. The live weight gain of kids in this experiment was also higher in kids of high-protein-fed does. It is thought that due to higher milk consumption and higher live weight gain in kids, the body length, wither height, and heart girth of kids was higher in the high-protein-fed group than in the others in this experiment.

In the current study, the survivability of kids was higher in the kids of medium- (14% CP) and high-protein (18% CP)-fed does, although the numbers in each treatment group were small. The survivability rate of kids was reduced from 71% at a high level of protein feeding to 62% at a low level of protein feeding of the dam during gestation. Chowdhury et al. [11] attributed the lower mortality rate of Black Bengal kids to the nutritional status of their dams and found that milk availability had a positive association with their thriftiness. An enhancement in birth weight and immune status with superior nutrition in the dams may help explain their better growth performance. A similar conclusion can be made from the results of the present study. 

Live weight and the composition of diet are the two main factors associated with carcass traits [35]. In our study, the higher protein content of the 18% CP diet enhanced growth performance, as reflected in the higher slaughter weight, hot carcass weight, and dressing percentage of their kids. The fat content of muscle tissue was also enhanced. Similarly, Qin et al. [36] reported that the supplementation of basal forage diets for weaned lambs with high-protein-content sea buckthorn pomace promoted intramuscular fat content but had no significant effect on other nutritional contents. Gómez et al. [37] stated that the energy/protein ratio in the diet influenced fatness in pigs. Wang et al. [38] revealed that dietary protein levels significantly increased fat content in muscle tissue in Tibetan sheep. In our study, it is suggested that high-protein intake may have induced the increased fat content in muscle tissue.

The expression level of the *H-FABP* gene was significantly higher in the kids from the high-protein-fed (18% CP) does than from the two lower-protein-fed groups. Li et al. [39] stated that high dietary protein (12.99% CP) levels positively influenced intramuscular fat deposition, leading to improved meat quality attributes, including tenderness and flavor, in Yunshang Black Goat. In cows, protein restriction during pregnancy reduced early collagen content in calf skeletal muscle and resulted in poor collagen remodeling in later stages of growth [40]. However, a diet high in crude protein (13% CP) for beef cattle may result in a higher marbling score and carcass yield than a diet low in protein (11% CP) [41]. A high-CP diet (14% CP) also induced a higher marbling score relative to a 12% CP diet in Hanwoo steers [42]. The *H-FABP* mRNA expression level in the high-energy group was significantly higher than that in the moderate- and low-energy groups in sheep [43]. According to Brandstetter et al. [44], *H-FABP* supplies long-chain fatty acids (LCFAs) as an important energy source for muscle growth and maintenance and also directs LCFAs towards fat storage within muscle fibers. 

Interestingly, the addition of single amino acids (leucine or arginine) increased intramuscular fat content in porcine muscle [45]. High-protein diets may provide essential amino acids that are precursors for the synthesis of lipids within muscle cells. This can alter metabolic pathways, promoting lipid synthesis within muscle cells, thereby increasing intramuscular fat content [46]. On the other hand, proteins can modulate the levels of hormones such as insulin and insulin-like growth factors (IGFs), which play critical roles in nutrient partitioning and adipogenesis. These hormones enhance the deposition of fat within muscle fibers rather than subcutaneous fat, contributing to higher intramuscular fat levels [46]. Duarte et al. [47] revealed that increasing the amount of maintenance feeding of pregnant Nellore cows by 1.5 times elevated the mRNA expression of *ZFP423*, *CEBPA*, and *PPARG*, which are key transcriptional regulators of adipogenesis, in the skeletal muscle of their calves. Practical measures such as a high plane of nutrition can modulate the differentiation of adipocyte precursor cells and the expression of genes related to this process, thereby increasing the development and deposition of intramuscular fat. In the current study, it is assumed that the higher expression level of the *H-FABP* gene was due to higher levels of protein in the diet, which provide essential amino acids that promote the synthesis of lipids and greater fat content in meat.

## 5. Conclusions

The results suggest that dietary protein levels during late pregnancy influences milk yield, lactation length, and post-partum anestrous in Black Bengal does and enhances the growth performance and survival rate of kids while influencing their hot carcass weight, dressing percentage, and fat content in their meat.

## Figures and Tables

**Table 1:** The ingredients and chemical composition of the experimental diets (DM basis).

Ingredients (Kg)	Protein Levels		
	High (18% CP)	Medium (14% CP)	Low (10% CP)
German grass	55.58	52.66	45.47
Wheat bran	10.32	14.92	8.63
Maize crushed	10.95	20.15	42.74
Soybean meal	22.15	11.27	2.16
Common salt	0.5	0.5	0.5
Dicalcium phosphate	0.5	0.5	0.5
Total	100	100	100
Chemical composition			
Estimated CP (%)	18	14	10
Estimated ME (MJ/kg DM)	10.0	10.0	10.0
NDF (%)	46.16	46.18	40.31
ADF (%)	29.59	28.42	24.34
Ca (%)	0.43	0.39	0.33
P (%)	0.53	0.52	0.44

ME and CP values of feed ingredients were taken from values published by Banerjee [12], Ranjan [13], Khan et al. [14], and Heuzé et al. [15]; NDF: neutral detergent fiber; ADF: acid detergent fiber.

**Table 2:** List of primers and primer sequences used for qPCR.

Primer Name	Primer Sequence (5′-3′)	Gene Bank Accession Number
*H-FABP*	FWD: GGTGGCCAATATGACCAAACC REV: TCAAGCTGGGAGTCGAGTTC	AY_466498.1
*β-actin*	FWD: CTCCCTGGAGAAGAGCTACG REV: GCAGGATTCCATGCCCAGG	NM_001314342.1

*β-Actin* was used as internal control for real-time PCR analysis.

**Table 3:** The effect of the maternal dietary protein levels in late pregnancy on the live weight changes of Black Bengal does before parturition (*n* = 4 per group).

Parameter	Protein Level		
	High (18% CP)	Medium (14% CP)	Low (10% CP)
Initial live weight (kg)	19.38 ± 1.58	19.45 ± 1.46	19.43 ± 2.28
Final live weight (kg)	22.45 ± 2.34	20.33 ± 1.53	19.53 ± 1.58
Live weight gain (kg)	3.08 ^a^ ± 0.30	0.88 ^b^ ± 0.68	0.10 ^b^ ± 0.00
Average daily feed intake (g/d)	980.59 ± 50.31	906.90 ± 18.53	886.21 ± 17.55

Means with uncommon superscripts in the same row differ significantly at the 5% level (*p* < 0.05).

**Table 4:** Effects of maternal dietary protein levels in late pregnancy on performance of Black Bengal does.

Parameter	Protein Level		
	High (18% CP)	Medium (14% CP)	Low (10% CP)
Number of does	4	4	4
Litter size (kids)	1.75 ± 0.25	1.50 ± 0.29	2.00 ± 0.00
Birth type (%)			
Single birth	25	50	-
Twin birth	75	50	100
Gender (%)			
Male kids	3 (42.86)	3 (50.00)	3 (37.50)
Female kids	4 (57.14)	3 (50.00)	5 (62.50)
Birth weight of kids (kg)			
Male kids	1.50 ± 0.06	1.23 ± 0.03	1.30 ± 0.06
Female kids	1.02 ± 0.17	1.13 ± 0.03	0.94 ± 0.16
Lactation length of does (d)	99.00 ^a^ ± 3.19	83.75 ^b^ ± 0.85	62.00 ^c^ ± 1.22
Post-partum heat period (d)	86.50 ^b^ ± 0.50	88.00 ^ab^ ± 0.71	89.67 ^a^ ± 1.45

Means with uncommon superscripts in the same row differ significantly at the 5% level (*p* < 0.05).

**Table 5:** Effects of maternal dietary protein levels during pregnancy on growth of kids.

Parameters	Diet (*n* = 5 per Group)				Gender of Kids Male (*n* = 3 per Group) and Female (*n* = 2 per Group)			Diet and Sex Interaction
	High (18% CP)	Medium (14% CP)	Low (10% CP)	*p*-Value	Male	Female	*p*-Value	*p*-Value
Weaning weight (kg)	5.74 ^a^	5.20 ^ab^	4.10 ^b^	0.039	5.36 ^a^	4.35 ^b^	0.034	0.271
Final live weight (kg)	8.72 ^a^	7.82 ^b^	6.98 ^c^	0.002	8.97 ^a^	6.15 ^b^	0.000	0.354
Total live weight gain (kg)	7.16 ^a^	6.38 ^b^	5.66 ^c^	0.003	7.51 ^a^	4.73 ^b^	0.000	0.656
Average daily gain (g/d)	31.61 ^a^	28.48 ^a^	24.07 ^b^	0.002	33.5 ^a^	19.84 ^b^	0.000	0.524

Means with uncommon superscripts in the same row differ significantly at the 5% level (*p* < 0.05).

**Table 6:** Effects of maternal dietary protein levels on blood parameters of kids during pre-weaning period (*n* = 5 per group).

Parameter	Protein Level		
	High (18% CP)	Medium (14% CP)	Low (10% CP)
Glucose (mmol/L)	3.60 ± 0.03	3.59 ± 0.32	3.58 ± 0.23
Total protein (g/dL)	7.85 ± 0.30	7.70 ± 0.17	7.58 ± 0.24
Albumin (g/dL)	3.91 ± 0.15	3.81 ± 0.20	3.90 ± 0.15
Globulin (g/dL)	3.95 ± 0.24	3.89 ± 0.14	3.68 ± 0.21
Triglyceride (mg/dL)	22.58 ± 2.31	24.61 ± 1.94	26.68 ± 5.68

**Table 7:** Effects of maternal dietary protein levels on blood parameters of kids during post-weaning period (*n* = 5 per group).

Parameter	Protein Level		
	High (18% CP)	Medium (14% CP)	Low (10% CP)
Glucose (mmol/L)	3.71 ± 0.05	3.70 ± 0.31	3.69 ± 0.31
Total protein (g/dL)	8.18 ± 0.35	7.90 ± 0.16	7.73 ± 0.22
Albumin (g/dL)	3.99 ± 0.18	3.91 ± 0.21	3.96 ± 0.16
Globulin (g/dL)	4.19 ± 0.29	3.99 ± 0.12	3.77 ± 0.19
Triglyceride (mg/dL)	24.19 ± 2.26	26.30 ± 2.30	29.15 ± 6.50

**Table 8:** Effects of maternal dietary protein levels on survivability rate of Black Bengal kids.

Parameter	Protein Level		
	High (18% CP)	Medium (14% CP)	Low (10% CP)
No. of does	4	4	4
No. of kids born	7	6	8
No. of kids died	2	1	3
No. of kids survived	5	5	5
Survivability (%)	71	83	62

**Table 9:** Effects of maternal dietary protein levels on carcass characteristics of Black Bengal kids (*n* = 3 per group).

Parameter	Protein Level		
	High (18% CP)	Medium (14% CP)	Low (10% CP)
Slaughter weight (kg)	10.07 ^a^ ± 0.15	8.80 ^b^ ± 0.35	8.03 ^b^ ± 0.12
Hot carcass weight (kg)	4.70 ^a^ ± 0.31	3.87 ^b^ ± 0.15	3.27 ^b^ ± 0.07
Dressing percentage (%)	46.63 ^a^ ± 2.42	43.98 ^ab^ ± 1.25	40.67 ^b^ ± 0.59
Offal			
Heart (kg)	0.05 ± 0.01	0.04 ± 0.00	0.04 ± 0.00
Liver (kg)	0.26 ± 0.00	0.25 ± 0.01	0.25± 0.01
Kidney (kg)	0.05 ± 0.00	0.05 ± 0.00	0.04 ± 0.00
Lung (kg)	0.17 ± 0.01	0.16 ± 0.01	0.16 ± 0.00
Spleen (kg)	0.03 ± 0.00	0.03 ± 0.00	0.029 ± 0.00
Stomach (kg)	2.87 ± 0.07	2.77 ± 0.03	2.83 ± 0.03
Empty gut (kg)	1.43 ± 0.07	1.41 ± 0.04	1.36 ± 0.06
Gut fill (kg)	1.37 ± 0.12	1.33 ± 0.03	1.53 ± 0.09

Means with uncommon superscripts in the same row differ significantly at the 5% level (*p* < 0.05).

**Table 10:** Effects of maternal dietary protein levels on nutrient composition of *longissimus* muscle of Black Bengal kids (*n* = 3 per group).

Parameter (%)	Protein Level		
	High (18% CP)	Medium (14% CP)	Low (10% CP)
Dry matter	25.24 ± 0.92	25.13 ± 0.83	24.62 ± 2.14
Crude protein	23.77 ± 0.84	23.16 ± 0.59	21.92 ± 1.52
Ether extract	2.51 ^a^ ± 0.12	1.75 ^b^ ± 0.23	1.46 ^b^ ± 0.06
Ash	1.01 ± 0.10	0.98 ± 0.19	1.10 ± 0.15

Means with uncommon superscripts in the same row differ significantly at the 5% level (*p* < 0.05).

## Data Availability

Data are contained within the article.
